# Pre-transplant depression decreased overall survival of patients receiving allogeneic hematopoietic stem cell transplantation: a nationwide cohort study

**DOI:** 10.1038/s41598-020-71208-2

**Published:** 2020-09-17

**Authors:** Sheng-Min Wang, Sung-Soo Park, Si-Hyun Park, Nak-Young Kim, Dong Woo Kang, Hae-Ran Na, Jong Wook Lee, Seunghoon Han, Hyun Kook Lim

**Affiliations:** 1 Department of Psychiatry, Yeouido St. Mary’s Hospital, College of Medicine, The Catholic University of Korea, 10, 63-ro, Yeongdeungpo-gu, Seoul, Republic of Korea; 2 Department of Hematology, Seoul St. Mary’s Hospital, College of Medicine, The Catholic University of Korea, Seoul, Republic of Korea; 3 Department of Pharmacology, College of Medicine, The Catholic University of Korea, 222, Banpo-daero, Seocho-gu, Seoul, Republic of Korea; 4 Department of Psychiatry, Seoul St. Mary’s Hospital, College of Medicine, The Catholic University of Korea, Seoul, 06591 Republic of Korea

**Keywords:** Neurology, Oncology, Risk factors, Signs and symptoms

## Abstract

Studies investigating association of depression with overall survival (OS) after allogeneic hematopoietic stem cell transplantation (allo-HSCT) yielded conflicting results. A nationwide cohort study, which included all adult patients [n = 7,170; depression group, 13.3% (N = 956); non-depression group, 86.7% (N = 6,214)] who received allo-HSCT from 2002 to 2018 in South Korea, analyzed risk of pre-transplant depression in OS of allo-HSCT. Subjects were followed from the day they received allo-HSCT, to occurrence of death, or last follow-up day (December 31, 2018). Median age at allo-HSCT for depression and non-depression groups were 50 and 45 (*p* < 0.0001), respectively. Two groups also differed in rate of females (depression group, 55.8%; non-depression group, 43.8%; *p* < 0.0001) and leukemia (depression group, 61.4%; non-depression group, 49.7%; *p* < 0.0001). After a median follow-up of 29.1 months, 5-year OS rate was 63.1%. Cox proportional-hazard regression evaluated an adjusted risk of post-transplant mortality related to depression: OS decreased sequentially from no depression (adjusted hazard ratio [aHR] = 1) to pre-transplant depression only (aHR = 1.167, CI: 1.007–1.352, *p* = 0.04), and to having both depression and anxiety disorder (aHR = 1.202, CI: 1.038–1.393, *p* = 0.014) groups. Pre-transplant anxiety (anxiety only) did not have significant influence in OS. Additional medical and psychiatric care might be necessary in patients who experienced depression, especially with anxiety, before allo-HSCT.

## Introduction

Allogeneic hematopoietic stem cell transplantation (allo-HSCT) provides curative therapy for patients with malignant and nonmalignant hematologic disorders^1,2^. However, allo-HSCT is a highly aggressive and demanding medical supports with a significant risk of mortality^3^. Besides primary hematologic disorder and disease relapse after transplantation, transplant-related mortality is an important obstacle influencing treatment outcome of allo-HSCT^4^. Thus, careful assessment of risk and accurate prediction of mortality are necessary before undertaking allo-HSCT^5^.

Depression is a common comorbidity of oncological and chronic hematologic diseases^6^. Studies showed that more than 20% of patients receiving allo-HSCT experience clinically significant depression before undergoing transplantation^7^. Besides its negative impact on the quality of life, depression is also associated with poor treatment survival outcome in patients receiving allo-HSCT^8^. Thus, most stem cell transplantation centers require patients to receive a comprehensive psychiatric evaluation as a part of the pre-transplant process^9^. Numerous prognostic scoring systems recognizes depression as an important risk factor of post-transplant mortality^10^. The Hematopoietic cell transplantation-specific comorbidity index (HCT-CI), one of the most important prognostic scoring systems in allo-HSCT, acknowledged that depression increases post-transplant mortality with hazard ratio of 1.8^11^.

Studies investigating the impact of pre-transplant depression on overall survival (OS) of patients receiving allo-HSCT have yielded conflicting results^12,13^. A study comprising of 123 patients suggested that existing diagnosis of depression before allo-HSCT were not related to post-transplant OS^14^. In contrast, a more recent study showed that, among 190 patients receiving allo-HSCT, depression before transplantation predicted higher 1-year (hazard ratio [HR] = 2.59; *p* = 0.014) and 3-year mortality (HR = 2.04; 95% *p* = 0.041)^15^. Thus, studies containing larger cohorts are needed to confirm temporal association between pre-transplant depression and OS of allo-HSCT.

A recent large sized cohort (n = 7,433) study, using data from the Center for International Blood and Marrow Transplant Research (CIBMTR), showed that pre-transplant depression was associated with lower OS (HR = 1.13; 95% confidence interval [CI], 1.04–1.23; *p* = 0.004)^16^. However, the study neither used a validated measurement nor diagnostic assessment to define pre-transplant depression. Rather, the study merely used retrospective chart review of a single question reported in the CIBMTR database; was there “clinically significant depression requiring treatment?” Inevitably, the diagnosis of depression was prone to reporter bias. To overcome this bias, the study excluded transplant centers having more than 10 transplants without reports of both depressed and nondepressed patients to the registry. By doing so, selection bias became another important limitation. Co-occurrence of anxiety disorder and depression are very common in patients receiving allo-HSCT^17^. By neglecting possible overlapping impact of pre-transplant anxiety, it is still uncertain whether depression truly increased post-transplant mortality independent from the anxiety. Whether anxiety disorder causes additive risk in post-transplantation mortality risk among patients with depression history is another important issue needing investigation. Patients with leukemia are known to have a higher post-transplant mortality than patients with non-malignant hematologic disease^18,19^. Thus, subgroup analysis comprising more homogenous group, leukemia patients only, are needed to address detrimental effect of pre-transplant depression in OS of allo-HSCT. Lastly, CIBMTR is an US based registry, so studies containing a large sample size must also be repeated in diverse ethnicities to confirm impact of pre-transplant depression in outcomes of allo-HSCT.

To fill in this important gap, we conducted a nationwide longitudinal cohort study using the National Healthcare Insurance Service (NHIS) database containing data of majority of allo-HSCTs performed in South Korea (hereafter “Korea”). We first replicated previous findings by investigating association of pre-transplant depression in the long-term survival of allo-HSCT in Asian population (Koreans). Beyond replication, we extended previous work by analyzing independent risk of pre-transplant depression, separating possible overlapping effect of anxiety, in OS of allo-HSCT. Whether negative impact of pre-transplant depression in allo-HSCT is augmented by co-morbid anxiety was also evaluated. In addition, sub-group analysis of patients with leukemia was conducted to confirm risk of pre-transplant depression in allo-HSCT among higher risk group. Lastly, landmark analyses were performed at 100 days post-transplant to exclude patients at high risk of early mortality post-transplant and to evaluate the impact of prognostic variables on OS of patients still alive at these time points.

## Results

### Participant characteristics

Between January 2002 and December 2018, a total of 7,170 adult patients who received allo-HSCT were included in this study (Table 1). 13.3% (N = 956) were diagnosed with and visited hospital due to depression before transplantation whereas 86.7% (N = 6,214) did not. The mean duration of depression and anxiety before transplantations were 609.4 ± 569.3 days (median: 414.5 days) and 710.9 ± 587.5 days (median: 607 days) respectively. The median age at allo-HSCT was higher for depression group than non-depression group. Depression group had more subjects who were females, had leukemia, received peripheral blood as stem cell source, and had previous non-hematologic malignancies. Likewise, diagnosis of anxiety, hypertension, diabetes, dyslipidemia, chronic obstructive pulmonary disease, and cerebro- or cardiovascular disease were also higher in depression group than in non-depression group.

**Table 1:** Basal demographical and clinical characteristics of adult patients who received allo-HSCT.

Variables	Total cohort (N = 7,170)	History of depression before allogeneic-HSCT		
		Yes (N = 956)	No (N = 6,214)	*p*-value
**Median age at transplant, year (range)**	45	50	44	< 0.0001
Age ≤ median ( 45 years old)	3,664 (51.1%)	359 (37.6%)	3,305 (53.2%)	
Age > median ( 45 years old)	3,506 (48.9%)	597 (62.4%)	2,909 (46.8%)	
**Gender**				< 0.0001
Male, no (%)	3,917 (54.6%)	423 (44.2%)	3,494 (56.2%)	
**Hematologic disease**				< 0.0001
Leukemia. no (%)	3,676 (51.3%)	587 (61.4%)	3,089 (49.7%)	
Hodgkin lymphoma, no (%)	23 (0.3%)	6 (0.6%)	17 (0.3%)	
Non-Hodgkin lymphoma, no (%)	374 (5.2%)	75 (7.8%)	299 (4.8%)	
Multiple myeloma, no (%)	144 (2.0%)	31 (3.2%)	113 (1.8%)	
MPN, no (%)	68 ( 0.9%)	11 (1.2%)	57 (0.9%)	
MDS, no (%)	663 (9.2%)	68 (7.1%)	595 (9.6%)	
Aplastic anemia, no (%)	464 (6.4%)	41 (4.3%)	423 (6.8%)	
Unclassified, no (%)	1758 (24.5%)	137 (14.3%)	1621 (26.1%)	
**Stem cell source**				< 0.0001
Bone marrow, no (%)	747 (10.4%)	77 (8.1%)	670 (10.8%)	
Peripheral blood, no (%)	4,752 (66.3%)	730 (76.4%)	4,022 (64.7%)	
Unclassified, no (%)	1671 (23.3%)	149 (15.6%)	1522 (24.5%)	
**Anxiety**				< 0.0001
No, no (%)	5,752 (80.2%)	482 (50.4%)	5,270 (84.8%)	
Yes, no (%)	1,418 (19.8%)	474 (49.6%)	944 (15.2%)	
**Previous non-hematologic malignancy**				< 0.0001
No, no (%)	6,482 (90.4%)	818 (85.6%)	5,664 (91.1%)	
Yes, no (%)	688 (9.6%)	138 (14.4%)	550 (8.9%)	
**Hypertension**				< 0.0001
No, no (%)	5,168 (72.1%)	585 (61.2%)	4,583 (73.8%)	
Yes, no (%)	2002 (27.9%)	371 (38.8%)	1631 (26.2%)	
**Diabetes**				< 0.0001
No, no (%)	5,284 (73.7%)	600 (62.8%)	4,684 (75.4%)	
Yes, no (%)	1886 (26.3%)	356 (37.2%)	1,530 (24.6%)	
**Dyslipidemia**				< 0.0001
No, no (%)	3,736 (52.1%)	364 (38.1%)	3,372 (54.3%)	
Yes, no (%)	3,434 (47.9%)	592 (61.9%)	2,842 (45.7%)	
**COPD**				< 0.0001
No, no (%)	6,834 (95.3%)	887 (92.8%)	5,947 (95.7%)	
Yes, no (%)	336 (4.7%)	69 (7.2%)	267 (4.3%)	
**CVD**				< 0.0001
No, no (%)	6,905 (96.3%)	895 (93.6%)	6,010 (96.7%)	
Yes, no (%)	265 (3.7%)	61 (6.4%)	204 (3.3%)	

*COPD* Chronic obstructive pulmonary disease; *CVD* Cerebro- or cardiovascular disease; *HSCT* Hematopoietic stem cell transplantation; *MDS* Myelodysplastic syndrome; *MPN* myeloproliferative neoplasm.

### Survival outcome: total cohort (N = 7,170)

**Figure 1:**
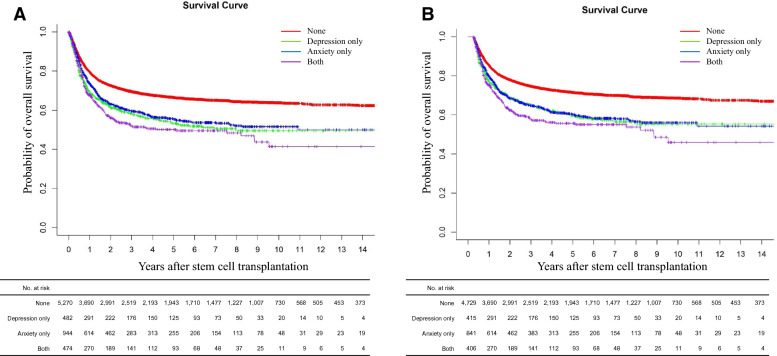
Overall survival outcome difference according to depression and or anxiety history; (**A**) in total cohort (**B**) in landmark cohort at the designated 100 days post-transplant.

After a median follow-up of 29.1 months (range 0 to 207), the estimated overall survival (OS) rates at 5 years were 63.1% (supplementary Fig. 1). Supplementary Table 1 summarizes the unadjusted analyses comparing allo-HCT outcomes between depression and non-depression group. Patients with pre-transplant depression (depression only group) and anxiety (anxiety only group) had lower 5 year-OS than those who neither had depression nor anxiety (none group), and 5 year-OS was the lowest in patients having both depression and anxiety (both depression and anxiety group) (Fig. 1A).

The factors found to be significant in the univariable analysis were all included in the final multivariable analysis (Table 2). The post-transplantation OS decreased sequentially from none group (adjusted hazard ratio [aHR] = 1) to depression only group (aHR = 1.167, CI: 1.007–1.352, *p* = 0.04), and to both depression and anxiety group (aHR = 1.202, CI: 1.038–1.393, *p* = 0.014). However, anxiety only group did not have significant influence in OS.

**Table 2:** Multivariable analysis of factors affecting overall survival of allo-HSCT in all patients (N = 7,170).

Variable	Hazard ratio (95% CI)	*p*-value
**Age**		
Age ≤ median (45 years old)	1	
Age > median (45 years old)	1.249 (1.143–1.367)	< 0.0001
**Sex**		
Female	1	
Male	1.151 (1.060–1.250)	< 0.0001
**Pre-transplantation depression**		
No without anxiety (none)	1	
Yes without anxiety (depression only)	1.167 (1.007–1.352)	0.040
No with anxiety (anxiety only)	1.038 (0.927–1.162)	0.519
Yes with anxiety (depression and anxiety)	1.202 (1.038–1.393)	0.014
**Hematologic disease**		
Aplastic anemia	1	
MDS/MPN	2.083 (1.611–2.693)	< 0.0001
Leukemia/all lymphomas/Multiple myeloma	3.063 (2.435–3.853)	< 0.0001
**Stem cell source**		
Bone marrow	1	
Peripheral blood	1.457 (1.268–1.675)	< 0.0001
**Previous other malignancy**		
No	1	
Yes	1.188 (1.052–1.343)	0.006
**Hypertension**		
No	1	
Yes	1.260 (1.152–1.377)	< 0.0001
**Diabetes**		
No	1	
Yes	1.080 (0.985–1.183)	0.099
**Dyslipidemia**		
No	1	
Yes	1.030 (0.985–1.123)	0.509
**COPD**		
No	1	
Yes	1.065 (0.893–1.270)	0.486
**CVD**		
No	1	
Yes	1.438 (1.206–1.715)	< 0.0001

*COPD* Chronic obstructive pulmonary disease; *CVD* Cerebro- or cardiovascular disease; *HSCT* Hematopoietic stem cell transplantation; *MDS* Myelodysplastic syndrome; *MPN* myeloproliferative neoplasm.

### Survival outcome: landmark cohort (N = 6,391)

Among 7,170 adult patients who received allo-HSCT, 6,391 patients were alive at the designated 100 days landmark and were included in the landmark analysis (supplementary Fig. 2 for OS). In line with total cohort, depression only group and anxiety only group had lower 5 year-OS, with the both depression and anxiety group having the lowest 5 year-OS (Fig. 1B). The factors found to be significant in the univariable analysis (supplementary Table 2) were all included in the final multivariable analysis (Table 3). However, the multivariable analysis of the landmark cohort showed that the OS after transplantation was significantly lower only in both depression and anxiety group (aHR = 1.253, CI: 1.061–1.479, *p* < 0.001), and neither depression only nor anxiety only group showed statistical significance.

**Table 3:** Multivariable analysis of factors affecting overall survival of allo-HSCT in landmark cohort (N = 6,391).

Variable	Hazard ratio (95% CI)	*p*-value
**Age**		
Age ≤ median (45 years old)	1	
Age > median (45 years old)	1.265 (1.144–1.400)	< 0.0001
**Sex**		
Female	1	
Male	1.196 (1.089–1.312)	< 0.0001
**Pre-transplantation depression**		
None	1	
Depression only	1.155 (0.975–1.369)	0.0947
Anxiety only	1.083 (0.955–1.229)	0.2149
Depression and anxiety	1.253 (1.061–1.479)	< 0.001
**Hematologic disease**		
Aplastic anemia	1	
MDS/MPN	3.006 (2.156–4.191)	< 0.0001
Leukemia/all lymphomas/Multiple myeloma	4.785 (3.530–6.487)	< 0.0001
**Stem cell source**		
Bone marrow	1	
Peripheral blood	1.416 (1.215–1.651)	< 0.0001
**Previous other malignancy**		
No	1	
Yes	1.109 (0.960–1.281)	0.1597
**Hypertension**		
No	1	
Yes	1.208 (1.091–1.338)	< 0.0001
**Diabetes**		
No	1	
Yes	1.104 (0.995–1.225)	0.0609
**Dyslipidemia**		
No	1	
Yes	1.040 (0.943–1.148)	0.4536
**COPD**		
No	1	
Yes	1.009 (0.822–1.240)	0.9288
**CVD**		
No	1	
Yes	1.448 (1.180–1.778)	< 0.0001

*COPD* Chronic obstructive pulmonary disease; *CVD* Cerebro- or cardiovascular disease; *HSCT* Hematopoietic stem cell transplantation; *MDS* Myelodysplastic syndrome; *MPN* myeloproliferative neoplasm.

### Subgroup analysis of patients with leukemia

**Figure 2:**
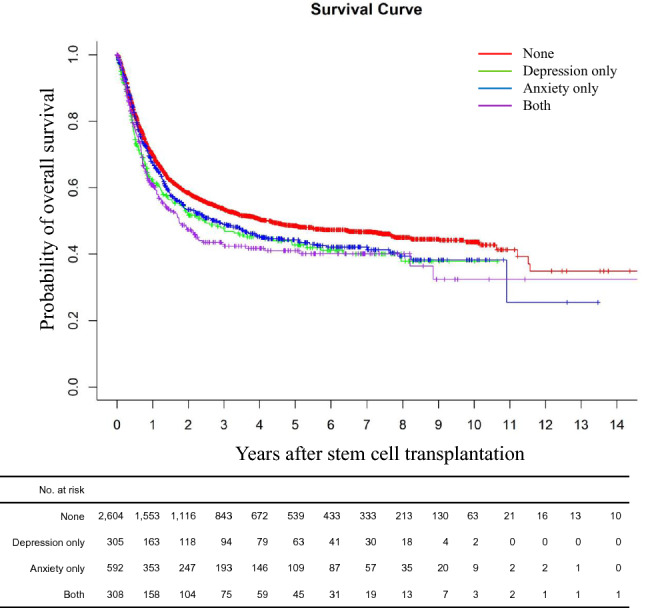
Overall survival outcome difference according to depression and or anxiety history in patients with leukemia.

In the multivariable analysis of total and landmark cohort, hematologic disease had the highest aHR (2.083–3.063 for total cohort and 3.006–4.785 for landmark cohort) (Tables 2, 3). In order to completely eliminate disease as a co-factor, we conducted sub-group analysis in patients with a more homogenous group, leukemia only. A total of 3,809 patients received allo-HSCT due to leukemia, and their baseline demographic and clinical data are summarized in Table 4, and OSs are shown in supplementary Fig. 3. Same trend was noted for univariable analyses (supplementary Table 3) and multivariable model (Table 5). Unadjusted analyses showed that depression only group, anxiety only group, and both depression and anxiety group all had lower 5 year-OS than none group (Fig. 2). Once again, the association was not detected for anxiety only group after adjusting for significant variables. In line with the total cohort, OS decreased sequentially from none group (aHR = 1) to depression only group (aHR = 1.220, CI: 1.031–1.444, *p* = 0.021), and to both depression and anxiety disorder group (aHR = 1.238, CI: 1.046–1.465, *p* = 0.0132).

**Table 4:** Baseline demographical and clinical characteristics of adult patients who received allo-HSCT due to leukemia.

Variables	Cohort (N = 3,809)	History of depression		*p*-value
		Yes (N = 613)	No (N = 3,196)	
**Median age at transplant, year (range)**	47 (18–74)	49(18–72)	46(18–74)	< 0.0001
Under 47, no (%)	1809 (52.2%)	344 (56.1%)	1,465 (45.8%)	
Over 47, no (%)	2000 (47.8%)	269 (43.9%)	1731 (54.2%)	
**Gender**				
Male, no (%)	2055 (54.0%)	255 (41.6%)	1,800 (56.3%)	< 0.0001
**Anxiety**				
No	2,909 (76.4%)	305 (49.8%)	2,604 (81.5%)	
Yes	900 (23.6%)	308 (50.2%)	592 (18.5%)	
**Stem cell source**				0.114
Bone marrow, no (%)	469 (12.3%)	62 (10.1%)	407 (12.7%)	
Peripheral blood, no (%)	3,246 (85.2%)	534 (87.1%)	2,712 (84.9%)	
Unclassified, no (%)	94 (2.5%)	17 (2.8%)	77 (2.4%)	
**Previous non-hematologic malignancy**				0.010
No, no (%)	3,422 (89.8%)	528 (86.1%)	2,894 (90.6%)	
Yes, no (%)	387 (10.2%)	85 (13.9%)	302 (9.4%)	
**Hypertension**				0.005
No, no (%)	1,224 (32.1%)	378 (61.7%)	2,207 (69.1%)	
Yes, no (%)	1,224 (32.1%)	235 (38.3%)	989 (30.9%)	
**Diabetes**				0.002
No, no (%)	2,684 (70.5%)	400 (65.3%)	1,446 (45.2%)	
Yes, no (%)	1,125 (29.5%)	213 (34.7%)	912 (28.5%)	
**Dyslipidemia**				< 0.0001
No, no (%)	1674 (43.9%)	228 (37.2%)	1,446 (45.2%)	
Yes, no (%)	2,135 (56.1%)	385 (62.8%)	1,750 (54.8%)	
**COPD**				0.022
No, no (%)	3,618 (95.0%)	570 (93.0%)	3,048 (95.4%)	
Yes, no (%)	191 (5.0%)	43 (7.0%)	148 (4.6%)	
**CVD**				0.079
No, no (%)	3,643 (95.6%)	579 (94.5%)	3,064 (95.9%)	
Yes, no (%)	166 (4.4%)	34 (5.5%)	132 (4.1%)	

*COPD* Chronic obstructive pulmonary disease; *CVD* Cerebro- or cardiovascular disease; *HSCT* Hematopoietic stem cell transplantation; *MDS* Myelodysplastic syndrome; *MPN* myeloproliferative neoplasm.

**Table 5:** Multivariable analysis of factors affecting overall survival of patients receiving allogeneic hematopoietic stem cell transplantation due to leukemia (all leukemia, N = 3,809).

Variable	Hazard ratio (95% CI)	*p*-value
**Age**		
Age ≤ median (47 years old)	1	
Age > median (47 years old)	1.185 (1.068–1.314)	< 0.0001
**Sex**		
Female	1	
Male	1.144 (1.040–1.259)	0.006
**Pre-transplantation depression**		
Neither depression nor anxiety (none)	1	
Depression without anxiety (depression only)	1.220 (1.031–1.444)	0.021
Anxiety without depression (anxiety only)	1.107 (1.031–1.259)	0.1244
Both depression and anxiety (depression and anxiety)	1.238 (1.046–1.465)	0.0132
**Stem cell source**		
Bone marrow	1	
Peripheral blood	1.498 (1.285–1.746)	< 0.0001
**Previous other malignancy**		
No	1	
Yes	1.161 (0.997–1.352)	0.0545
**Hypertension**		
No	1	
Yes	1.162 (1.046–1.291)	0.005
**Diabetes**		
No	1	
Yes	0.995 (0.893–1.109)	0.9288
**Dyslipidemia**		
No	1	
Yes	1.055 (0.954–1.167)	0.2961
**Chronic obstructive pulmonary disease**		
No	1	
Yes	1.033 (0.832–1.281)	0.7704
**Cerebro- or cardiovascular disease**		
No	1	
Yes	1.510 (1.224–1.861)	< 0.0001

## Discussion

Our results showed that pre-transplant depression, independent from anxiety, decreased OS of adult patients receiving allo-HSCT. We also showed that co-occurrence of depression with anxiety disorder further decreased post-transplant OS, but pre-transplantation anxiety disorder did not independently increase mortality after allo-HSCT. In terms of sub-group analysis, same trend was found in patients who received allo-HSCT due to leukemia. The post-transplantation OS decreased sequentially from neither depression nor anxiety group (none; aHR = 1) to having depression only (aHR = 1.202), and then to having both depression and anxiety (aHR = 1.231). However, in the landmark analysis at 100 days post-transplantation, OS after transplantation was significantly lower only in patients having both depression and anxiety (aHR = 1.253).

Numerous studies suggested association of pre-transplantation depression with lower OS in allo-HSCT^15,20–22^. However, only one large sized longitudinal cohort study conducted in the US using CIBMTR data showed that depression history decreases the OS with aHR of 1.13^16^. To the best of our knowledge, this is the largest nationwide cohort study conducted in Asia, or outside US, showing the risk of pre-transplantation depression in OS of allo-HSCT. We replicated previous research and showed that pre-transplant depression decreases OS with aHR of 1.167, which was very similar to the HR reported by El-Jawahri et al. By carefully sub-classifying patients according to their anxiety disorder history, we were able to eliminate overlapping impact of pre-transplant anxiety from that of depression. Thus, our findings more specifically confirmed that pre-transplant depression independently decreases OS of allo-HSCT regardless of anxiety disorder. We also extended previous work by showing that OS decrement was more pronounced in patients who had both depression and anxiety before allo-HSCT (aHR = 1.202).

Interestingly, we did not detect significant association between anxiety disorder and OS of allo-HSCT. Anxiety is a frequent response to threat, so it is commonly found in patients having severe medical conditions^23^. Consequently, it is difficult to accurately distinguish normal anxiety or adjustment reaction from that of true morbid anxiety^24^. Thus, patients having anxiety reaction could have been over-diagnosed as having anxiety disorder in our cohort, which might have led to decreased significance in OS. In line with this hypothesis, patients in our cohort had twice more history of anxiety than depression. Previous study also showed that patients having pre-transplantation depression more concordantly experienced clinically significant depression even after transplantation, whereas patients having pre-transplant anxiety tended to experience anxiety symptoms less continuously^17,25,26^. Thus, depression might had longer impact than anxiety in OS of patients receiving allo-HSCT. In addition, landmark analyses performed at 100 days post-transplant, which excluded patients at high risk of early mortality post-transplant, showed that OS was significantly lower only in patients who had both depression and anxiety disorder. Thus, prospective studies which include more complete predictors of mortality in allo-HSCT are needed to confirm complicated relationship among depression, anxiety, and survival outcome after allo-HSCT.

Our results have additional strengths. First, by conducing a nationwide cohort study, we included all adult patients who received allo-HSCT from 2002 to 2018. We were able to prevent selection bias and minimize recruitment setting effect, so our results have higher generalizability. Second, a more stringent definition was used to determine pre-transplant depression. Rather than using a single questionnaire or retrospective chart review, all patients with pre-transplant depression visited hospital due to exact diagnosis of depression [ICD-10 code for major depressive disorder (F32 × or F33 ×)]. Thus, we further prevented selection and reporting bias. Lastly, by conducting a subgroup analysis comprising of patients with leukemia only, we eliminated underlying hematologic disease as a possible co-factor and confirmed the negative impact of depression, and additive effect of anxiety, in post-transplant mortality.

The pathophysiological basis of pre-transplantation depression having a negative impact in OS of patients receiving allo-HSCT is still obscure. Nevertheless, our findings could be predicted by association of depression with immune system dysregulations^27^. Studies showed that depression is often accompanied by inflammatory diseases including irritable bowel syndrome, type 2 diabetes, arthritis and autoimmune disorders, which can activate the peripheral and central inflammatory response^28^. Furthermore, other studies illustrated that depression and acute graft-versus-host-disease (GVHD) share common pro-inflammatory reactions: increment of inflammatory cytokines (i.e. tumor necrosis factor-alpha) and shifting toward T helper 1 response system^29,30^. Likewise, past study showed that pre-HCT depression increased the incidence of acute GVHD^16^. Poor treatment adherence due to depression is another important cause of higher mortality^31^. However, additional prospective studies focusing on biological mechanism are needed to define common pathway linking depression, and anxiety, with that of post-transplantation mortality in patients receiving allo-HSCT.

Several limitations should also be noted. First, occurrence of depression was based on clinical diagnosis with hospital visit records rather than using objective depressive scale measurements. As a result, we were unable to assess mortality difference depending on the depression severity. Since the depression severity is not known, some patients in the non-depression group may have had depression but have been undiagnosed. Second, we were also unable to investigate antidepressant effect, or treatment effect, in the risk of mortality. Likewise, depression episodes after transplantation were not considered. Thus, the effect of depression recurrence or chronicity in the post-transplant mortality were not studied. Third, whether time to diagnosis from depression and anxiety or duration of their illnesses influenced OS were not analyzed. Fourth, unbalanced baseline characteristics between depression group and non-depression group could harbor possible bias. Fifth, the cause of death, and relapse related and non-relapse related mortality according to depression were not investigated. In addition, other important clinical factors such as GVHD and infection episodes after transplantation, pre-transplantation conditioning, performance score, remission status at time of transplant, type of donor, smoking history, and alcohol status were not considered. Lastly, although Korea has a mandatory public health insurance system providing a comprehensive medical coverage to all residents of Korea, socioeconomic status could still have influenced the OS.

In conclusion, pre-transplant depression, independent from anxiety, decreased OS of adult patients receiving allo-HSCT. The mortality risk was higher in patients who had both depression and anxiety than patients having neither. Same trend was noted in patients who received allo-HSCT due to leukemia. Thus, our results suggest that patients having depression, especially both depression and anxiety, before allo-HSCT have a higher risk of post-transplantation mortality needing more intensive care.

## Methods

### Data source and study population

The Korean National Health Insurance Service (KNHIS) is a mandatory public health insurance system of Korea and offers comprehensive medical coverage to all residents of Korea. Data were acquired from the KNHIS database, which contains all medical claims for the population covered under the KNHIS. The database has been widely used in various epidemiological studies and is described in detail elsewhere^32–34^.

Adult patients (≥ 18 years) who underwent allo-HSCT between 2002 and 2018 were identified using KNHIS database procedure codes X5061 for bone marrow and X5063 for peripheral blood allo-HSCT. For those who received second allo-HSCT, we used first allo-HSCT data as their baseline values. As a result, nearly all allo-HSCTs performed in Korea during the study period were included in the analysis. Comorbidities including hypertension, diabetes mellitus, chronic obstructive pulmonary disease, cerebro- or cardiovascular disease, and anxiety disorder were also extracted using International Classification of Diseases, Tenth Revision (ICD-10) codes. This study was approved by the institutional review board of Seoul St. Mary’s Hospital, Seoul, Korea (KC19ZNSI0396). Consent from individual subjects were not needed because the study used publicly open, anonymous data.

### Outcome variable and statistical analysis

The primary endpoint of this study was OS difference after allo-HSCT according to pre-transplant depression history. In order to utilize more conservative definition of depression, patients having ICD-10 code for major depressive disorder (F32 × or F33 ×) and visited hospital due to the same code within five years prior to the allo-HSCT was defined as having pre-transplant depression. Difference between the two groups (depression group and non-depression group) in baseline demographic and clinical characteristics were compared using student’s-T test for continuous variables and Chi-squared test for categorical variables.

To investigate independent risk of pre-transplant depression, and prevent overlapping impact of anxiety, we also identified patients who were diagnosed with and visited hospital due to anxiety disorder (F30 ×) before allo-HSCT. Thus, patients were subdivided in to four groups; no history of depression and anxiety disorder (none), depression history without anxiety (depression only), anxiety disorder without depression (anxiety only), and having both depression and anxiety disorder (both depression and anxiety).

Retrospective cohorts were followed from the day they received allo-HSCT to the occurrence of death or the last follow-up day (December 31, 2018), whichever came first. The OS rate represents the proportion of patients who were alive at the specified time after the date of transplantation and was associated with death due to any cause. Cox proportional-hazard regression evaluated risk of post-transplant mortality related to depression. All risk factors with *p* < 0.10 in univariable analysis were included in the single multivariable model. Thereafter, the factors found to be significant in this single multivariable model with depression variable were all included in the final multivariable model. For all statistical analysis, we used R statistical software (ver. 3.1.1, R Foundation for Statistical Computing, Vienna, Austria, 2012).

### Ethical approval

All procedures contributing to this work comply with the ethical standards of the relevant national and institutional committees on human experimentation and with the Helsinki Declaration of 1975, as revised in 2008.

## Supplementary information

Supplementary Information.
