# Supplementary material for: Pre-transplant depression decreased overall survival of patients receiving allogeneic hematopoietic stem cell transplantation: a nationwide cohort study
Source: Sci Rep. 2020 Sep 17;10:15265. doi: 10.1038/s41598-020-71208-2 (PMC7499172; doi:10.1038/s41598-020-71208-2)

Pre-transplant Depression Decreased Overall Survival of Patients Receiving Allogeneic Hematopoietic Stem Cell Transplantation: A Nationwide Cohort Study

Short title: Depression Decreases Survival of Stem Cell Transplantation

Sheng-Min Wang^1*^, Sung-Soo Park^2*^, Si-Hyun Park^3^, Nak-Young Kim^1^, Dong Woo Kang^4^, Hae-Ran Na^1^, Jong Wook Lee^2^, Seunghoon Han^3#^, and Hyun Kook Lim^1#^

^1^Department of Psychiatry, Yeouido St. Mary’s Hospital, College of Medicine, The Catholic University of Korea, Seoul, Korea

^2^Department of Hematology, Seoul St. Mary’s Hospital, College of Medicine, The Catholic University of Korea, Seoul, Korea

^3^Department of Pharmacology, College of Medicine, The Catholic University of Korea, Seoul, Korea

^4^Department of Psychiatry, Seoul St. Mary’s Hospital, College of Medicine, The Catholic University of Korea, Seoul, Korea

^*^ Sheng-Min Wang and Sung-Soo Park equally contributed as first authors.

^#^ Seunghoon Han and Hyun Kook Lim equally contributed as corresponding authors.

Address for Correspondence:

Seunghoon Han, MD, PhD

Department of Pharmacology, College of Medicine, the Catholic University of Korea, 222,

Banpo-daero, Seocho-gu, Seoul, Korea [06591]

Tel: +82-2-2258-7326

Fax: +82-2-2258-7876

Email: [waystolove@catholic.ac.kr](mailto:waystolove@catholic.ac.kr)

And

Hyun Kook Lim, MD, PhD

Department of Psychiatry, Yeouido St. Mary’s Hospital, College of Medicine, The Catholic University of Korea 10, 63-ro, Yeongdeungpo-gu, Seoul, Republic of Korea

Tel; +82-2-3779-1048

Fax: +82-2-780-6577

Email: [drblues@catholic.ac.kr](mailto:drblues@catholic.ac.kr)


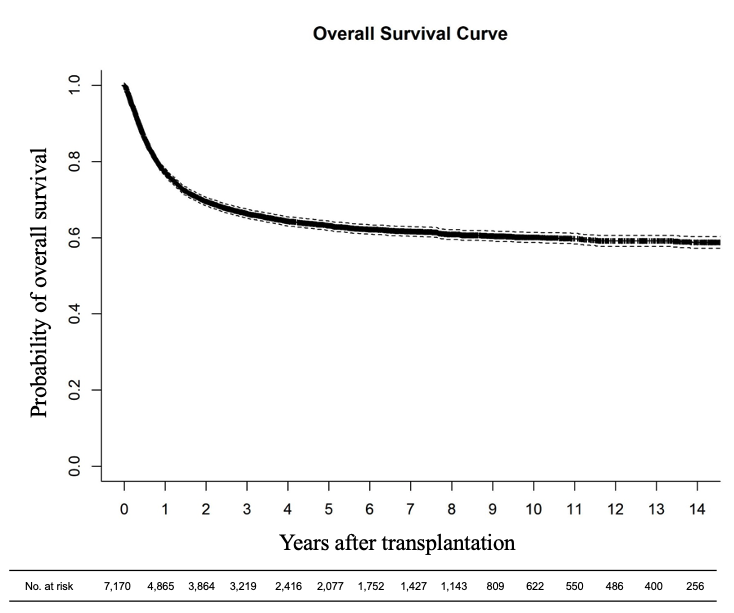


Supplementary figure 1. Overall survival outcome of total cohort


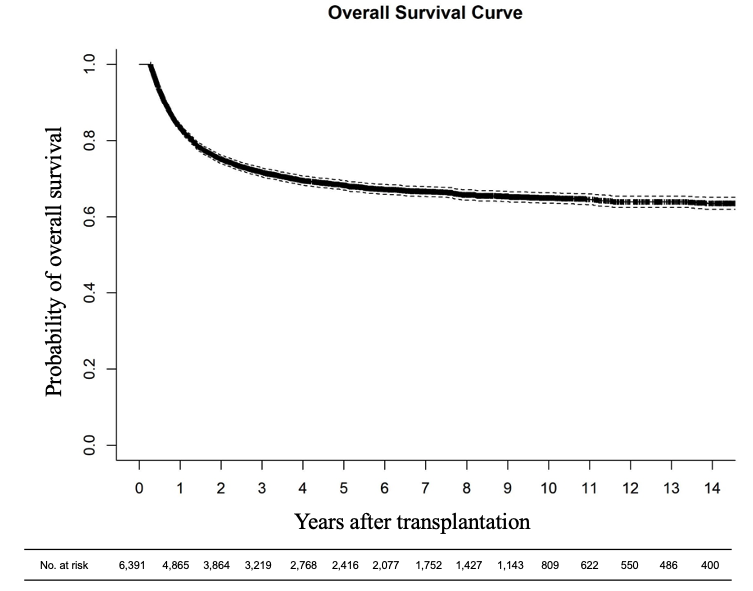


Supplementary figure 2. Overall survival based on a landmark analysis at 100 days post-transplant


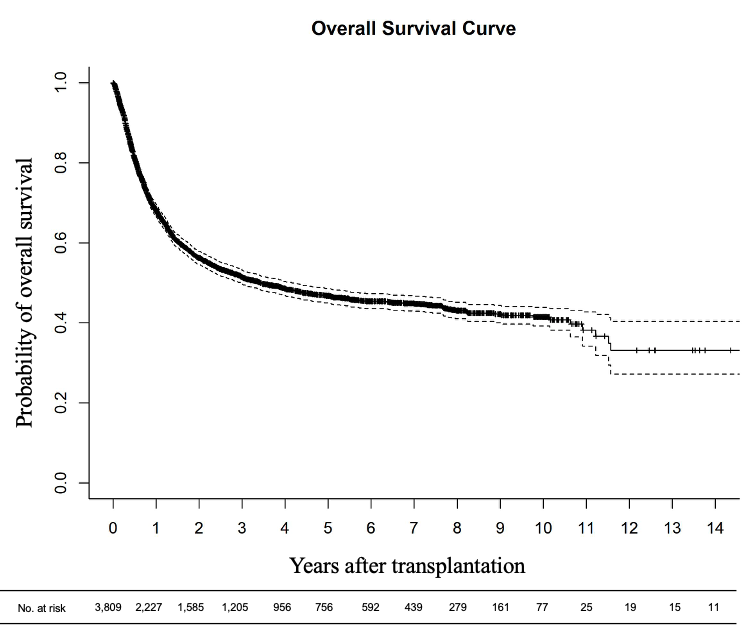


Supplementary figure 3. Overall survival outcome of patients who received transplantation due to leukemia

**­­Supplementary Table 1.** Univariable analysis of factors affecting overall survival of allogeneic HSCT in all patients (N=7170)

| Variable | N (7,170) | OS at 5 years (95% CI) | Median OS, months (95% CI) | *p-value* |
| --- | --- | --- | --- | --- |
| **Age** |  |  |  | < 0.0001 |
| Age ≤median (45 years old) | 3,664 | 70.8% (69.3 – 72.4) | NA |  |
| Age > median (45 years old) | 3,506 | 54.5% (52.6 – 56.4) | 113.8 (91.8 – 178.8) |  |
| **Sex** |  |  |  | < 0.0001 |
| Female | 3,253 | 66.4% (64.6 – 68.2) | NA |  |
| Male | 3,917 | 60.3% (58.7 – 62.1) | NA |  |
| **Psychiatrics stratus** |  |  |  | < 0.0001 |
| None | 5,270 | 66.5% (65.1 – 67.9) | NA |  |
| Depression only | 482 | 53.2% (48.4 – 58.5) | 95.5 (52,8 – NA) |  |
| Anxiety only | 944 | 55.3% (51.9 – 59.0) | 131.1 (84.2 – NA) |  |
| Both depression and anxiety | 474 | 50.1% (45.2 – 55.5) | 61.3 (26.5 – NA) |  |
| **Hematologic disease** |  |  |  | < 0.0001 |
| Leukemia | 3,809 | 47.0% (45.2 – 48.9) | 41.2 (35.7 – 49.9) |  |
| Hodgkin lymphoma | 20 | 41.1% (24.6 – 68.7) | 15.4 (7.7 – NA) |  |
| Non-Hodgkin Lymphoma | 267 | 41.0% (35.3 – 47.5) | 24.8 (13.2 – 43.9) |  |
| Multiple myeloma | 121 | 32.4% (24.2 – 43.3) | 32.1 (18.3 – 46.7) |  |
| MPN | 68 | 52.7% (40.2 – 69.0) | 93.8 (18.7 – NA) |  |
| MDS | 663 | 58.0% (53.8 – 62.5) | NA (108.6 – NA) |  |
| Aplastic anemia | 464 | 82.7% (79.2 – 86.4) | NA |  |
| Unclassified | - | - |  |  |
| **Stem cell source** |  |  |  | < 0.0001 |
| Bone marrow | 747 | 70.3% (67.0 – 73.7) | NA |  |
| Peripheral blood | 4,752 | 50.1% (48.5 – 51.8) | 61.1 (48.4 – 83.6) |  |
| Unclassified | - | - |  |  |
| **Previous other malignancy** |  |  |  | < 0.0001 |
| No | 6,482 | 64.5% (63.3 – 65.8) | NA |  |
| Yes | 688 | 49.1% (44.9 – 53.7) | 53.5 (32.2 – 94.4) |  |
| **Hypertension** |  |  |  | < 0.0001 |
| No | 5,168 | 67.7% (66.3 – 69.1) | NA |  |
| Yes | 2,002 | 50.7% (48.3 – 53.3) | 63.4 (49.9 – 92.3) |  |
| **Diabetes** |  |  |  | < 0.0001 |
| No | 5,284 | 67.1% (65.7 – 68.5) | NA |  |
| Yes | 1,886 | 51.4% (48.8 – 54.0) | 62.8 (53.1 – 93.0) |  |
| **Dyslipidemia** |  |  |  | < 0.0001 |
| No | 3,736 | 69.9% (68.4 – 71.5) | NA |  |
| Yes | 3,434 | 54.6% (52.7 – 56.6) | 122.0 (87.1 – NA) |  |
| **COPD** |  |  |  | < 0.0001 |
| No | 6,834 | 63.6% (62.4 – 64.9) | NA |  |
| Yes | 336 | 51.3% (45.3 – 58.2) | 93.0 (39.0 – NA) |  |
| **CVD** |  |  |  | < 0.0001 |
| No | 6,905 | 64.0% (62.8 – 65.3) | NA |  |
| Yes | 265 | 36.7% (30.2 – 44.6) | 20.3 (15.9 – 35.9) |  |
| COPD: Chronic obstructive pulmonary disease; CVD: Cerebro- or cardiovascular disease; HSCT: Hematopoietic stem cell transplantation; MDS: Myelodysplastic syndrome; MPN: myeloproliferative neoplasm. | | | | |

**Supplementary Table 2.** Univariable analysis of factors affecting overall survival of allogeneic HSCT in landmark cohort (N=6,391)

| Variable | N (6,391) | OS at 5 years (95% CI) | Median OS, months (95% CI) | *p-value* |
| --- | --- | --- | --- | --- |
| **Age** |  |  |  | < 0.0001 |
| Age ≤ median (45 years old) | 3,349 | 75.1% (73.5 – 76.7) | NA |  |
| Age > median (45 years old) | 3,042 | 60.1% (58.1 – 62.1) | 183.8 (161.2 – NA) |  |
| **Sex** |  |  |  | < 0.0001 |
| Female | 2,925 | 71.6% (69.8 – 73.4) | NA |  |
| Male | 3,466 | 65.4% (63.6 – 67.1) | NA |  |
| **Psychiatrics stratus** |  |  |  | < 0.0001 |
| None | 4,729 | 71.5% (70.1 – 72.9) | NA |  |
| Depression only | 415 | 59.4% (54.3 – 64.9) | NA (95.5 – NA)) |  |
| Anxiety only | 841 | 60.0% (56.5 – 63.8) | 183.8 (131.1 – NA) |  |
| Both depression and anxiety | 406 | 55.6% (50.5 – 61.3) | 106.4 (61.3 – NA) |  |
| **Hematologic disease** |  |  |  | < 0.0001 |
| Leukemia | 3,320 | 51.5% (49.6 – 53.5) | 70.7 (55.8 – 93.6) |  |
| Hodgkin lymphoma | 17 | 42.9% (23.6 – 77.8) | 32.0 (9.5 – NA) |  |
| Non-Hodgkin Lymphoma | 209 | 49.3% (41.7 – 58.2) | 56.4 (31.5 – NA) |  |
| Multiple myeloma | 100 | 41.7% (31.4 – 55.3) | 38.9 (32.1 – 69.9) |  |
| MPN | 56 | 59.9% (46.4 – 77.3) | 93.8 (33.6 – NA) |  |
| MDS | 577 | 63.9% (59.5 – 68.5) | NA |  |
| Aplastic anemia | 421 | 89.6% (86.5 – 92.8) | NA |  |
| Unclassified | - | - |  |  |
| **Stem cell source** |  |  |  | < 0.0001 |
| Bone marrow | 704 | 74.1% (70.9 – 77.6) | NA |  |
| Peripheral blood | 4,087 | 55.7% (54.0 – 57.5) | NA (98.8 – NA) |  |
| Unclassified | - | - |  |  |
| **Previous other malignancy** |  |  |  | < 0.0001 |
| No | 5,817 | 69.3% (68.0 – 70.6) | NA |  |
| Yes | 574 | 56.4% (51.9 – 61.3) | 162.9 (77.2 – NA) |  |
| **Hypertension** |  |  |  | < 0.0001 |
| No | 4,695 | 72.1% (70.7 – 73.5) | NA |  |
| Yes | 1,696 | 57.0% (54.4 – 59.8) | 138.4 (98.5 – NA) |  |
| **Diabetes** |  |  |  | < 0.0001 |
| No | 4,765 | 71.9% (70.5 – 73.3) | NA |  |
| Yes | 1,626 | 56.9% (54.2 – 59.7) | 123.6 (91.8 – NA) |  |
| **Dyslipidemia** |  |  |  | < 0.0001 |
| No | 3,419 | 74.4% (72.9 – 76.0) | NA |  |
| Yes | 2,972 | 60.1% (58.0 – 62.1) | NA |  |
| **COPD** |  |  |  | < 0.0001 |
| No | 6,116 | 68.6% (67.4 – 69.9) | NA |  |
| Yes | 275 | 57.8% (51.3 – 65.1) | NA (93.0 – NA) |  |
| **CVD** |  |  |  | < 0.0001 |
| No | 6,177 | 69.0% (67.8 – 70.3) | NA |  |
| Yes | 214 | 42.7% (35.4 – 51.5) | 32.3 (21.2 – 75.6) |  |
| COPD: Chronic obstructive pulmonary disease; CVD: Cerebro- or cardiovascular disease; HSCT: Hematopoietic stem cell transplantation; MDS: Myelodysplastic syndrome; MPN: myeloproliferative neoplasm. | | | | |

**Supplementary Table 3.** Univariable analysis of factors affecting overall survival of patients who received allo-HSCT due to leukemia (all leukemia, N=3,809)

| Variable | N (3,809) | OS at 5 years (95% CI) | Median OS, months (95% CI) | *p-value* |
| --- | --- | --- | --- | --- |
| **Age** |  |  |  | < 0.0001 |
| Age ≤median (47 years old) | 2,000 | 52.0% (49.6 – 54.5) | 87.8 (55.3 - 131.1) |  |
| Age > median (47 years old) | 1,809 | 40.6% (38.0 – 43.4) | 25.1 (21.7 - 32.6) |  |
| **Sex** |  |  |  | 0.026 |
| Female | 1,754 | 49.1% (46.5 – 51.8) | 49.7 (38.0 - 92.2) |  |
| Male | 2,055 | 44.7% (42.3 – 47.3) | 35.9 (28.9 - 47.0) |  |
| **Psychiatrics stratus** |  |  |  | 0.0014 |
| None | 2,604 | 48.5% (46.4 – 50.8) | 50.9 (40.6 - 66.0) |  |
| Depression only | 305 | 42.7% (36.8 – 49.5) | 29.5 (19.5 - 59.0) |  |
| Anxiety only | 592 | 44.3% (40.0 – 49.0) | 32.7 (22.4 - 47.1) |  |
| Both depression and anxiety | 308 | 41.0% (35.2 – 47.8) | 20.9 (15.3 - 35.4) |  |
| **Stem cell source** |  |  |  | < 0.0001 |
| Bone marrow, no (%) | 469 | 60.3% (55.9 – 65.1) | NA (98,6 - NA) |  |
| Peripheral blood, no (%) | 3,246 | 44.1% (42.1 – 46.2) | 34.6 (28.9 - 40.6) |  |
| Unclassified | 94 | - |  |  |
| **Non-psychiatric comorbidities** |  |  |  |  |
| **Previous other malignancy** |  |  |  | 0.0015 |
| No | 3,422 | 47.3% (45.4 – 49.3) | 44.3 (36.9 - 54.0) |  |
| Yes | 387 | 41.8% (36.3 – 48.2) | 22.8 (16.5 - 40.5) |  |
| **Hypertension** |  |  |  | < 0.0001 |
| No | 2,585 | 49.5% (47.4 – 51.8) | 55.3 (43.2 - 84.2) |  |
| Yes | 1,224 | 60.7% (37.6 – 44.1) | 23.8 (20.4 - 34.9) |  |
| **Diabetes** |  |  |  | 0.0049 |
| No | 2,684 | 48.1% (46.0 – 50.3) | 47.2 (39.2 - 61.1) |  |
| Yes | 1,125 | 43.4% (40.1 – 46.9) | 32.1 (23.3 - 41.7) |  |
| **Dyslipidemia** |  |  |  | 0.0066 |
| No | 1,674 | 49.1% (46.5 – 51.8) | 52.4 (39.2 - 87.8) |  |
| Yes | 2,135 | 44.7% (42.2 – 47.3) | 36.1 (29.5 - 45.9) |  |
| **COPD** |  |  |  | 0.21 |
| No | 3,618 | 46.9% (45.1 – 48.8) | 41.5 (35.8 - 50.7) |  |
| Yes | 191 | 43.0% (35.2 – 52.6) | 25.4 (16.7 - 93.0) |  |
| **CVD** |  |  |  | < 0.0001 |
| No | 3,643 | 47.5% (45.7 – 49.4) | 45.2 (37.3 - 55.3) |  |
| Yes | 166 | 28.0% (20.4 – 38.2) | 14.3 (10.6 - 18.8) |  |
| COPD: Chronic obstructive pulmonary disease; CVD: Cerebro- or cardiovascular disease; HSCT: Hematopoietic stem cell transplantation; MDS: Myelodysplastic syndrome; MPN: myeloproliferative neoplasm | | | | |


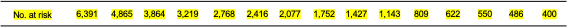

Supplement: Supplementary file 1 — Supplementary Information. [file 41598_2020_71208_MOESM1_ESM.docx]
